# Real-World Glycemic Improvements with Hybrid Closed Loop Pumps in Youth with Type 1 Diabetes

**DOI:** 10.1155/2023/6621706

**Published:** 2023-10-05

**Authors:** Maite E. Del Valle Rolón, Elizabeth A. Brown, Risa M. Wolf

**Affiliations:** Department of Pediatrics, Division of Endocrinology, Johns Hopkins University School of Medicine, Baltimore, USA

## Abstract

**Objective:**

The use of hybrid closed-loop insulin delivery systems, specifically the t:slim X2 insulin pump with Control IQ (CIQ), has demonstrated improvement in glycemic control in clinical trials and real-world settings. We sought to describe changes in glycemic control with use of CIQ in minority and nonminority youth. *Research Design and Methods*. This was a retrospective study of youth with type 1 diabetes (T1D) using CIQ over a 12-month period. Medical record data, pump data, and hemoglobin A1c (HbA1c) were collected from the visit prior to starting CIQ and at each clinic visit up to 12 months after starting CIQ. Continuous glucose monitor (CGM) data and HbA1c trajectory over time were compared to baseline and between minority and nonminority youth.

**Results:**

The study included 136 patients of whom 21 were minority youth (non-Hispanic Black and Hispanic), 50% were male, with median age of 13.3*y*, and median diabetes duration of 4.9*y*. After starting CIQ, baseline median HbA1c for the nonminority group decreased from 7.8% to 7.1% (*p* < 0.001), baseline median HbA1c for minority youth decreased from 9.8% to 7.8% (*p*=0.03), and the percentage of patients meeting target HbA1c <7% increased from 26% to 45%. Both nonminority and minority youth had a significant increase in time in range and decrease of average CGM glucose (*p* < 0.05).

**Conclusions:**

HbA1c levels decreased in both minority and nonminority youth within 12 months of starting CIQ, and more patients reached the HbA1c target of less than 7%. Disparities in HbA1c between minority and nonminority youth remained and additional studies are warranted to improve this.

## 1. Introduction

Diabetes is one of the most common chronic diseases in youth [1]. Over the last two decades, there has been an increase in incidence of type 1 diabetes (T1D) among US youth, especially among racial/ethnic minorities [2]. Glycemic control, commonly measured by levels of hemoglobin A1c (HbA1c), is essential to minimize acute and chronic complications [3, 4]. Although HbA1c target goal should be individualized in each patient, the American Diabetes Association recommends that an HbA1c less than 7% is appropriate for most pediatric patients [5]. However, evidence shows that glycemic control tends to vary between racial and ethnic subgroups, and non-Hispanic Black (NHB) youth are more likely to have higher HbA1c levels, higher risk for complications, and higher mortality rates than their white peers [4, 6].

Diabetes technology including continuous glucose monitor (CGM), insulin pumps, and a combination of both, have become an important element in the management of diabetes [5, 7–9]. The use of technology has improved quality of life, reduced complications such as hypoglycemia, and has led to higher glucose monitoring satisfaction [8, 10]. One of the more recent technologies is automated insulin delivery systems, or hybrid closed-loop systems (HCLS), where an algorithm automatically adjusts insulin delivery to maintain glucose levels in target range although still requiring the user to bolus for meals. These HCLS have shown significant improvement in glycemic control without increasing hypoglycemia, both in clinical trials and in real world settings, as well as improvement in psychosocial outcomes [9, 11–15].

It has been shown that NHB youth have higher HbA1c, more diabetic ketoacidosis (DKA) events, and more severe hypoglycemic events when compared to non-Hispanic White (NHW) and Hispanic populations [16]. There are also significant disparities in use of CGM and insulin pumps between NHB, Hispanic, and White patients [16]. Although diabetes technology use is associated with improved glycemic parameters, there is minimal representation of minority populations in CGM and pump trials, thereby limiting translatability of results to all individuals with diabetes [17, 18]. In this study, we aimed to evaluate a diverse population of youth with T1D utilizing the t:slim X2 insulin pump and Dexcom G6 CGM with Control-IQ (CIQ) Technology to determine if the use of this system in the real-world setting improves glycemic control in both minority and nonminority youth. We hypothesized that use of a HCLS would increase the number of patients reaching the target HbA1c.

## 2. Materials and Methods

This was a retrospective study of pediatric patients with T1D using the t:slim X2 insulin pump with CIQ Technology at the Johns Hopkins Pediatric Diabetes Center from two sites (Johns Hopkins Hospital and Mount Washington Pediatric Hospital). Data were collected from August 6, 2019 until April 27, 2022. The protocol was approved by the Johns Hopkins institutional review board according to the Declaration of Helsinki and with a waiver of consent.

Patients meeting the following criteria were included: age <25, diagnosis of T1D, and using CIQ hybrid closed loop technology on a t:slim insulin pump with Dexcom G6. Data were obtained from the participants' medical records from the diabetes visit encounter prior to starting CIQ and from each subsequent diabetes visit up to 12 months after starting CIQ. Since CIQ became available during the COVID-19 pandemic, a number of patients had virtual visits during this time, without HbA1c data available, and thus data were collected for up to 12 months after initiation of CIQ in order to capture HbA1c data.

Baseline data collected for each patient included race, ethnicity, sex, insurance type, date of diagnosis, initial HbA1c, and whether they were in DKA at diagnosis. Patients were further categorized as minority and nonminority for analysis. Patients that identified as Black or Hispanic were included in the minority cohort, and those that identified as NHW or Asian were classified as the nonminority cohort, with Asian youth included in this group due to similar HbA1c levels and glycemic control to NHW [19]. Before starting CIQ, patients' insulin delivery method and CGM use with associated glucose data were collected, in addition to body mass index (BMI) and BMI Z-score. Due to the increase in virtual visits during the COVID-19 pandemic, BMI and BMI *Z*-score were not available at every visit, and in those cases the BMI at the closest visit was used. At the follow-up visit after starting CIQ, similar data were collected, as well as time in use of the system was recorded. If the provider documented inconsistent use of CIQ in the medical record, these data were collected. HbA1c measurements were performed at the point of care for in-person clinic visits using the Alere Afinion AS100 Analyzer. Due to the COVID-19 pandemic, some visits were performed virtually, so if an HbA1c was measured within 45 of days of the appointment, it was attached to that visit. The CGM data, including percentage time use, average blood glucose value, and percentage of time above, at, or below target range were based on downloaded data from the prior 14 days. Target ranges were defined as: <54 mg/dL time below Range 2 (TBR2) 54–70 mg/dL time below Range 1 (TBR1), >70–180 mg/dL time in range (TIR), >180–250 mg/dL time above Range 1 (TAR1), and >250 mg/dL time above Range 2 (TAR2). A small number of patients (*n* = 4) had different targets set in the *T*-slim application for some visits, so the data from those visits could not be used and were excluded from the analysis.

The primary outcome was the change in HbA1c during the first 12 months after starting CIQ among minority and nonminority youth. Secondary outcomes were the percentage time spent in target glucose range (70–180 mg/dL), consistent use of CGM and CIQ, and percentage of patients meeting HbA1c <7%.

### 2.1. Statistical Analysis

A priori power analysis was conducted to calculate the required sample size to achieve 80% power with a type 1 two-tailed error rate of 5%. Standard deviation of HbA1c was assumed to be 0.9% points, based upon a sample of youth with T1D and a history of CGM use [20]. Variability between HbA1c pairs was specified at 75%. A sample size of 15 or more subjects per group was required in order to detect a mean paired decrease of 0.5% points in HbA1c after initiating CIQ. To detect a between group difference of 0.5% in the mean minority versus nonminority groups requires 31 subjects and 170 subjects, respectively, assuming a cohort that is 15% minority.

Categorical variables are described using frequencies and percentages. Comparisons between minority and nonminority groups were made using the *χ*^2^ test or the Fisher's Exact test, for variables with expected frequencies in any group of less than 5. The Shapiro–Wilks test was used to assess the normality distribution of continuous variables. Normally distributed variables were described by mean and standard deviation and comparisons between groups used a two-sample *t* test. Non-normally distributed variables were described by median and interquartile range and comparisons between groups used a Wilcoxon rank-sum test. Wilcoxon signed-rank tests were used to assess unadjusted differences between baseline and last follow-up within 12 months of baseline. For subjects with missing follow-up data, Wilcoxon rank-sum test were calculated to show no significant differences in baseline data compared to those with follow-up. Multivariable analysis was conducted using linear mixed effects longitudinal models with a randomly varying intercept. The model tested change in HbA1c over time (30 days months) while controlling for public insurance, minority race, sex, age at baseline (years), duration of diabetes (years), and a binary variable flagging those with inconsistent use of CIQ. An interaction variable testing the difference in the trajectory of HbA1c between the minority and nonminority group when adjusting for confounding variables was tested for significance. The values *p* < 0.05 were considered statistically significant. Statistical analysis was generated using SAS software version 9.4 Copyright© 2020 SAS Institute Inc.

## 3. Results

The study included 136 youth, of whom 68 (50%) were male with a median age of 13.3*y* (IQR 10.2–15.5), and a median diabetes duration of 4.9*y* (IQR 2.3–8.0). As shown in Table 1, the nonminority group included 115 youth (111 White, 4 Asian), and 21 youth comprised the minority group (17 NHB and 4 Hispanic). At baseline, minority youth were more likely to have public insurance (52% versus 13%, *p*=0.0002) and a higher HbA1c (9.6% versus 7.8%, *p*=0.0004) compared to nonminority youth. At the time of diagnosis, there were no significant differences between the groups in age, HbA1c, and whether they presented in DKA at diagnosis.

Before starting CIQ, a significantly higher number of nonminority patients were using CGM compared to minority patients (96% versus 81%, *p*=0.02), but there was no difference in CGM parameters between those using CGM in each group. Although not significant, a greater proportion of nonminority youth were using insulin pumps prior to starting CIQ compared to minority youth (82% versus 62%, *p*=0.08). Of those who were already using insulin pumps, most of this cohort was using the t:slim insulin pump. While the BMI and BMI *Z*-score did trend higher in the minority group, the differences were not statistically significant.

**Figure 1:**
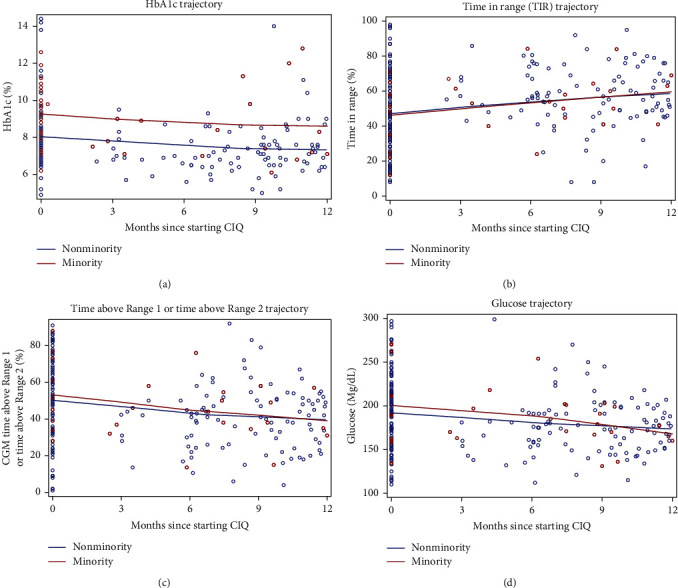
(a) HbA1c trajectory by group, (b) TIR trajectory by group, (c) time above Range 1 or time above Range 2 by group, and (d) glucose trajectory by group. *Note*: All plots show last follow up within 12 months for each subject. Loess fit with 1.25 smoothing parameter.

As shown in Table 2, there were 124 patients that had follow-up visits within 12 months of starting CIQ, with a significant decrease in HbA1c in both groups: from 7.8% to 7.1% (*p* < 0.001) in the nonminority group, and from 9.8% to 7.8% (*p*=0.03) in the minority group. Both groups had a significant decrease of average CGM glucose and increase in TIR (Figure 1). Among minority patients, six (29%) had documented inconsistent use of CIQ, while only nine (8%) of nonminority patients had documented inconsistent use of CIQ (*p*=0.01).

Prior to starting CIQ, 26% of patients had an HbA1c less than 7%; for nonminority patients, 30% met target HbA1c, and for minority patients 5% met target HbA1c. Within 12 months of starting CIQ, 46% overall met HbA1c target goal; 50% in the nonminority group and 18% in the minority group.

We assessed the longitudinal changes in HbA1c within 12 months of starting CIQ using a multivariable linear model and controlling for age, sex, duration of diabetes, race/ethnicity, insurance type, and consistent use of CIQ, as shown in Table 3. Adjusted analysis demonstrated that on average, HbA1c decreased by 0.09% points per 30 days of CIQ use (*p* < .0001). Those with documented inconsistent use of CIQ (*n* = 15) had higher baseline HbA1c, 0.98% points on average (*p*=0.017) and did not demonstrate a drop in the average HbA1c; instead HbA1c increased on average 0.063% points per 30 days (*p*=.00004). While minority patients, those with public insurance and females all had statistically significant higher unadjusted baseline HbA1c, when fit into an adjusted model also controlling for age and duration of diabetes, these differences were no longer statistically significant. Due to the lack of baseline CGM data for a number of patients, further multivariable analysis with TIR as an outcome were not feasible.

In an exploratory analysis, we sought to determine if use of CIQ closed the HbA1c gap between minority and nonminority groups. A Wilcoxon rank-sum test was performed and showed that there was insufficient evidence to demonstrate a narrowing of the gap in HbA1c between the two groups, with similarly no significant differences in the change in average glucose, TIR, and time above/below range between the groups.

## 4. Discussion

In this study we demonstrated that use of CIQ hybrid closed loop technology in the real-world setting was associated with a decrease in HbA1c in both nonminority and minority groups within 12 months after starting CIQ. Further, we showed that more patients overall (26%–46%) reached a target HbA1c of <7% with use of CIQ. Use of CIQ was associated with a 0.09% decrease in HbA1c for every 30 days of use over the first 12 months, and patients with consistent use of CIQ hybrid closed loop technology had greater improvements in HbA1c.

Since its approval by the FDA in 2019, the use of CIQ has been associated with an increase in time spent within the target glycemic range and a decrease in HbA1c without increasing hypoglycemic events [11–13, 15]. This is consistent with our findings, where after 12 months of using CIQ, TIR increased from 46% to 57% (*p* < 0.001) in the nonminority group, and from 51% to 60% (*p*=0.012) in the minority group. Time spent in hypoglycemia decreased in both groups as well.

Although HbA1c significantly decreased in both groups after starting CIQ, the analysis did not show a significant reduction in the HbA1c gap between both groups. Given the small sample size of the minority population, there was not enough power to show a difference in the rate of HbA1c improvement between the groups. Additionally, there was a higher baseline HbA1c before starting CIQ (9.8% for minority and 7.8% for nonminority) in the minority group. A longitudinal cohort study using the data of 1,313 youths in the search for Diabetes In Youth Study found a higher HbA1c in patients who had the highest HbA1c at baseline diagnosis, suggesting a relationship between the HbA1c in the first year of diagnosis and the longitudinal trends over time [4]. Further, CGM use is associated with improved glycemic control in youth with diabetes [8], and prior to starting CIQ, the minority group had fewer patients using CGM (81%) compared to the nonminority group (96%). This is consistent with the other studies, which have shown lower use of diabetes technology, both insulin pump and CGM, in NHB and Hispanic youth compared to the White youth [21]. A cross-sectional study of 300 young adults with T1D found large racial–ethnic disparities in CGM and insulin pump use with 72% of NHW patients using insulin pumps versus 39% of Hispanics and 18% NHB patients, and 71% of NHW patients using CGM versus 37% of Hispanics and 28% NHB patients [21]. The reason for this is likely multifactorial, including socioeconomic status, patient's therapy preference, patient–provider relationship, and provider implicit bias [6, 21, 22]. Although important, the use of technology alone does not seem to account for all disparities in glycemic control, as seen in a longitudinal cohort study of 978 youth and young adults where technology and self-management tools only accounted for 35% of the differences in HbA1c between both groups [23].

We also saw a direct correlation between consistent use of CIQ and improvement of HbA1c. This is similar to data from clinical trials that show that participants who spend more time in automated mode have higher TIR [12, 13, 24]. Interruptions to CGM use occur for various reasons, including when pharmacy supplies are interrupted, the device falls off early, or there are adhesive issues, and therefore affects patient's ability to use the HCLS [20]. Common barriers to use of diabetic devices include economic issues such as insurance coverage and cost of supplies, in addition to discomfort or dislike of wearing the device [25]. Encouraging patients to continuously wear CGM and helping them to avoid interruptions may help to increase consistent use of CIQ and thereby improve glycemic control.

A strength of our study is the diverse population of T1D youth. Most trials and real-world studies of CIQ were in predominantly NHW patient cohorts, and thus our data are more translatable in the diverse clinic settings. This study has several limitations, including the inherent limitations of a retrospective study. As such, and due to the COVID-19 pandemic, numerous appointments were done virtually and therefore HbA1c data were not always available in the association with CGM and CIQ data. Because not all patients were using a CGM before initiation of CIQ, we were limited in our ability to evaluate CGM data over time. Additionally, consistent use of CIQ was determined based on the chart review, which was limited by provider documentation of this variable. Further, while the cohort is diverse, the minority group was small, and thus potentially limited our findings.

In summary, we demonstrated that HbA1c improved within 12 months of starting CIQ in both minority and nonminority groups, with more patients meeting target HbA1c <7%. Similar improvements were seen among both minority and nonminority youth for CGM parameters including mean glucose and TIR, thus encouraging consistent use of CIQ could further improve overall glycemic control and long-term outcomes in this community. Future studies in diverse youth should investigate if utilizing HCLSs can mitigate gaps in HbA1c between minority and nonminority youth.

## Figures and Tables

**Table 1:** Baseline characteristics at last visit before Control IQ.

Variable	Nonminority (*n* = 115)	Minority (*n* = 21)	*p*-Value
Male, number (%)	56 (49%)	12 (57%)	0.48^a^
Age (*y*), median (Q1–Q3)	13.3 (9.9–15.5)	13.3 (11.7–16.4)	0.48^b^
Insurance (public), number (%)	15 (13%)	11 (52%)	0.0002^c^
BMI, mean (SD)	20.6 (4.3)	22.1 (5.3)	0.25^d^
BMI *Z*-score, mean (SD)	0.54 (0.8)	0.66 (1.0)	0.55^d^
Duration of diabetes (y), median (Q1–Q3)	5 (1.7–8)	4.7 (3.1–7.7)	0.62^b^
Age at diagnosis, mean (SD)	7.5 (3.9)	7.5 (3.5)	0.98^d^
HbA1c % at diagnosis, median (Q1–Q3), NM *n* = 98, M *n* = 16	11.6 (9.9–12.8)	10.9 (9.4–12.5)	0.33^b^
DKA at diagnosis, number (%), NM *n* = 107, M *n* = 18	54 (50%)	8 (44%)	0.64^a^
HbA1c %, median (Q1–Q3)	7.8 (6.9–8.8)	9.6 (8.3–10.5)	0.0004^b^
Insulin regimen			
MDI use, number (%)	21 (18%)	8 (38%)	0.0774^c^
Insulin pump use, number (%)	94 (82%)	13 (62%)	0.08^c^
T:slim pump (%)	81 (70%)	12 (57%)	0.23^a^
CGM data			
CGM use, number (%), NM *n* = 114	110 (96%)	17 (81%)	0.0204^c^
Percentage time CGM active, median (Q1–Q3), NM *n* = 43, M *n* = 6	93 (83.3–97.3)	94.9 (76.5–98.2)	0.65^b^
Average CGM glucose, mean (SD), NM *n* = 98, M *n* = 10	193.1 (43.6)	200.9 (50.7)	0.59^d^
CGM % TBR2, median (Q1–Q3), NM *n* = 71, M *n* = 8	0 (0–1)	0 (0–0.5)	0.46^b^
CGM % TBR1 median (Q1–Q3), NM *n* = 82, M *n* = 10	1 (0–3)	1.5 (1–3)	0.18^b^
CGM % time in range, median (Q1–Q3), NM *n* = 88, M *n* = 10	45 (27–65)	49.5 (25–58)	0.96^b^
CGM % TAR1, median (Q1–Q3), NM *n* = 86, M *n* = 9	28 (19–37)	23 (19–31)	0.48^b^
CGM % TAR2, median (Q1–Q3), NM *n* = 72, M *n* = 8	25 (9–37)	28 (14–55)	0.39^b^

^a^ *χ* ^2^ test; ^b^Wilcoxon rank-sum test; ^c^Fisher's exact test; ^d^Independent two-sample *t* test.

**Table 2:** Glycemic control within 12 months of starting Control IQ.

	Nonminority			Minority		
	Baseline median (IQR)	Follow-up median (IQR)	*p*-Value ^*∗*^	Baseline median (IQR)	Follow-up median (IQR)	*p*-Value ^*∗*^
HbA1c, NM *n* = 86, M *n* = 17	7.8 (2.1)	7.1 (1.3)	<0.0001	9.8 (1.7)	7.8 (1.9)	0.029
Percentage of TAR1 or TAR2, NM *n* = 76, *M* = 8	49.5 (40.7)	42 (25.7)	<0.0001	48 (38.5)	38 (12.25)	0.023
Time in range (TIR), NM *n* = 78, M *n* = 9	46 (41)	57 (25.5)	<0.0001	51 (33)	60 (10.3)	0.012
Percentage of TBR2 or TBR1, NM *n* = 76, *M* = 8	1 (4)	1 (1.9)	0.104	1.5 (1.5)	1.7 (1.1)	0.66
Glucose, NM *n* = 87, M *n* = 10	191 (60)	174 (43)	<0.0001	189.5 (100)	170.5 (26)	0.027

*Note*: Table 2 includes only those with both baseline and follow-up values, showing slight differences from Table 1, which includes all subjects.  ^*∗*^*p*-Values are calculated using the Wilcoxon signed-rank test.

**Table 3:** Longitudinal change in HbA1c for first 12 months after starting Control IQ.

	Unadjusted		Adjusted	
	Estimate (95% CI)	*p*-Value	Estimate (95% CI)	*p*-Value
Intercept			8.2 (7.35, 9.12)	<.0001
Time since baseline (30 day months)	−0.07 (−0.1, −0.04)	<0.0001	−0.09 (−0.12, -0.06)	<.0001
Minority (Black or Hispanic)	1.2 (0.52, 1.87)	0.001	0.61 (−0.07, 1.29)	0.08
Insurance (Public)	0.99 (0.37, 1.62)	0.002	0.61 (−0.01, 1.23)	0.05
Sex (female)	0.56 (0.05, 1.08)	0.03	0.45 (−0.004, 0.9)	0.05
Age at baseline (years)	0.002 (−0.07, 0.07)	0.95	−0.07 (−0.14, 0.01)	0.08
Duration of diabetes (years)	0.04 (−0.03, 0.1)	0.27	0.05 (−0.02, 0.12)	0.14
Documented inconsistent use of control IQ	1.83 (1.09, 2.57)	<0.0001	0.98 (0.17, 1.78)	0.02
Effect modification of inconsistent use on time since baseline	0.16 (0.07, 0.24)	0.0004	0.16 (0.07, 0.24)	0.0004

*Note*: Linear mixed-effects model with randomly varying intercept. All covariates are included in the adjusted model.

## Data Availability

The data used to support the findings of this study are included in the manuscript.

## References

[1] Pettitt D. J., Talton J., Dabelea D. (2014). Prevalence of Diabetes in U.S. youth in 2009: the SEARCH for diabetes in youth study. *Diabetes Care*.

[2] Divers J., Mayer-Davis E. J., Lawrence J. M. (2020). Trends in incidence of type 1 and type 2 diabetes among youths—selected counties and Indian reservations, United States, 2002–2015. *MMWR. Morbidity and Mortality Weekly Report*.

[3] diabetes control and complications trial research group (1994). Effect of intensive diabetes treatment on the development and progression of long-term complications in adolescents with insulin-dependent diabetes mellitus: diabetes control and complications trial. *The Journal of Pediatrics*.

[4] Kahkoska A. R., Shay C. M., Crandell J. (2018). Association of race and ethnicity with glycemic control and hemoglobin A1c levels in youth with type 1 diabetes. *JAMA Network Open*.

[5] American Diabetes Association Professional Practice Committee (2022). 14. Children and adolescents: *standards of medical care in diabetes—2022*. *Diabetes Care*.

[6] Agarwal S., Kanapka L. G., Raymond J. K. (2020). Racial–ethnic inequity in young adults with type 1 diabetes. *The Journal of Clinical Endocrinology & Metabolism*.

[7] Beck R. W., Bergenstal R. M., Laffel L. M., Pickup J. C. (2019). Advances in technology for management of type 1 diabetes. *The Lancet*.

[8] Laffel L. M., Kanapka L. G., Beck R. W. (2020). Effect of continuous glucose monitoring on glycemic control in adolescents and young adults with type 1 diabetes: a randomized clinical trial. *JAMA*.

[9] Bergenstal R. M., Nimri R., Beck R. W. (2021). A comparison of two hybrid closed-loop systems in adolescents and young adults with type 1 diabetes (FLAIR): a multicentre, randomised, crossover trial. *The Lancet*.

[10] Bergenstal R. M., Klonoff D. C., Garg S. K. (2013). Threshold-based insulin-pump interruption for reduction of hypoglycemia. *New England Journal of Medicine*.

[11] Messer L. H., Berget C., Pyle L. (2021). Real-world use of a new hybrid closed loop improves glycemic control in youth with type 1 diabetes. *Diabetes Technology & Therapeutics*.

[12] Ware J., Allen J. M., Boughton C. K. (2022). Randomized trial of closed-loop control in very young children with type 1 diabetes. *New England Journal of Medicine*.

[13] Brown S. A., Kovatchev B. P., Raghinaru D. (2019). Six-month randomized, multicenter trial of closed-loop control in type 1 diabetes. *New England Journal of Medicine*.

[14] Knoll C., Peacock S., Wäldchen M. (2022). Real-world evidence on clinical outcomes of people with type 1 diabetes using open-source and commercial automated insulin dosing systems: a systematic review. *Diabetic Medicine*.

[15] Pinsker J. E., Müller L., Constantin A. (2021). Real-world patient-reported outcomes and glycemic results with initiation of control-IQ technology. *Diabetes Technology & Therapeutics*.

[16] Willi S. M., Miller K. M., DiMeglio L. A. (2015). Racial–ethnic disparities in management and outcomes among children with type 1 diabetes. *Pediatrics*.

[17] Akturk H. K., Agarwal S., Hoffecker L., Shah V. N. (2021). Inequity in racial–ethnic representation in randomized controlled trials of diabetes technologies in type 1 diabetes: critical need for new standards. *Diabetes Care*.

[18] Phillip M., Nimri R., Bergenstal R. M. (2022). Consensus recommendations for the use of automated insulin delivery technologies in clinical practice. *Endocrine Reviews*.

[19] Khanolkar A. R., Amin R., Taylor-Robinson D., Viner R. M., Warner J. T., Stephenson T. (2016). Young people with type 1 diabetes of non-white ethnicity and lower socio-economic status have poorer glycaemic control in England and Wales. *Diabetic Medicine*.

[20] Giani E., Snelgrove R., Volkening L. K., Laffel L. M. (2017). Continuous glucose monitoring (CGM) adherence in youth with type 1 diabetes: associations with biomedical and psychosocial variables. *Journal of Diabetes Science and Technology*.

[21] Agarwal S., Schechter C., Gonzalez J., Long J. A. (2021). Racial–ethnic disparities in diabetes technology use among young adults with type 1 diabetes. *Diabetes Technology & Therapeutics*.

[22] Chalew S., Delamater A. M., Washington S. (2021). Can innovative technologies overcome HbA1c disparity for African–American youth with type 1 diabetes?. *Journal of Diabetes Science and Technology*.

[23] Kahkoska A. R., Pokaprakarn T., Alexander G. R. (2022). The impact of racial and ethnic health disparities in diabetes management on clinical outcomes: a reinforcement learning analysis of health inequity among youth and young adults in the SEARCH for diabetes in youth study. *Diabetes Care*.

[24] Brown S. A., Forlenza G. P., Bode B. W. (2021). Multicenter trial of a tubeless, on-body automated insulin delivery system with customizable glycemic targets in pediatric and adult participants with type 1 diabetes. *Diabetes Care*.

[25] Messer L. H., Tanenbaum M. L., Cook P. F. (2020). Cost, hassle, and on-body experience: barriers to diabetes device use in adolescents and potential intervention targets. *Diabetes Technology & Therapeutics*.

